# Constructing Biopolymer-Inorganic Nanocomposite through a Biomimetic Mineralization Process for Enzyme Immobilization

**DOI:** 10.3390/ma8095286

**Published:** 2015-09-09

**Authors:** Jian Li, Jun Ma, Tao Jiang, Yanhuan Wang, Xuemei Wen, Guozhu Li

**Affiliations:** 1 School of Material Science and Chemical Engineering, Tianjin University of Science and Technology, Tianjin 300457, China; E-Mails: lijian@tust.edu.cn (J.L.); majun_tust@sina.cn (J.M.); wangyanhuan_tust@sina.cn (Y.W.); 2 Tianjin Synthetic Material Research Institute, Tianjin 300220, China; E-Mail: xmwen@tju.edu.cn; 3 Key Laboratory for Green Chemical Technology of Ministry of Education, School of Chemical Engineering and Technology, Tianjin University, Tianjin 300072, China; E-Mail: liguozhu@tju.edu.cn

**Keywords:** biomimetic mineralization, nanocomposite, enzyme immobilization, silica, alginate, template

## Abstract

Inspired by biosilicification, biomimetic polymer-silica nanocomposite has aroused a lot of interest from the viewpoints of both scientific research and technological applications. In this study, a novel dual functional polymer, NH_2_-Alginate, is synthesized through an oxidation-amination-reduction process. The “catalysis function” ensures the as-prepared NH_2_-Alginate inducing biomimetic mineralization of silica from low concentration precursor (Na_2_SiO_3_), and the “template function” cause microscopic phase separation in aqueous solution. The diameter of resultant NH_2_-Alginate micelles in aqueous solution distributed from 100 nm to 1.5 μm, and is influenced by the synthetic process of NH_2_-Alginate. The size and morphology of obtained NH_2_-Alginate/silica nanocomposite are correlated with the micelles. NH_2_-Alginate/silica nanocomposite was subsequently utilized to immobilize β-Glucuronidase (GUS). The harsh condition tolerance and long-term storage stability of the immobilized GUS are notably improved due to the buffering effect of NH_2_-Alginate and cage effect of silica matrix.

## 1. Introduction

Recent years, polymer-silica nanocomposite have aroused a lot of interest from the viewpoints of both scientific research and technological applications [1,2,3]. By conventional chemical methods, the preparation of silica-based materials often involved extreme temperature, pressure, and pH [4]. In contrast, biosilicification processes in nature can overcome these disadvantages [5,6]. Silicatein [7] and silaffin [8] identified in marine sponges and diatoms, respectively, can *in vitro* induce the formation of silica from precursor under ambient conditions. Therefore, various synthetic polymers (polypeptides, polyamines) as well as biopolymers (proteins, polysaccharides) have been used as inducers to mimic the biosilicification processes occurring in living organisms [9,10,11,12]. The obtained polymer-silica nanocomposite is widely used in areas of bioreactors, biosensors, and bio-deliveries [12,13,14]. 

To mimic the biosilicification processes occurring in aqueous solution, the polymers should provide two functions in general. One is catalysis function, and the other is template function [15]. “Catalysis function” means that the polymer can accelerate the precipitation of silica from a precursor with low concentration. For either synthetic polymers or biopolymers, it has been proved that the catalysis function is related to their charge at neutral pH [10,16]. Previous research showed that proteins with isoelectric point (pI) > 7.0 (lysozyme, protamine, *etc.*) can induce the precipitation of silica. However, no precipitation was observed for those proteins with pI < 7.0. The total charge and cationic residues (–NH_2_, –SH) of the basic proteins play important roles for accelerating silica precipitation [17].

Template function is related to “microscopic phase separation” [18], one kind of self-assembly process which is generated by polymers when they contact with precursor [19]. Poulsen [20] proved that long-chain polyamines (LCPA) could undergo microscopic phase separation to form a microemulsion template, which significantly influenced the microstructure of polymer-silica nanocomposite. However, positively charged LCPA was incapable of precipitating silica individually unless polyanions or multivalent anions were added [20]. Brunner [21] explained the aggregation and phase separation of LCPA were formed by electrostatic interactions between the positively charged LCPA molecules and the negatively charged phosphate ions. Other multivalent anions [21,22] such as pyrophosphate, sulfate, or polyanion natSil-2 [20] were also capable of inducing the precipitation of silica from silicate-containing polyamine solutions.

In nature, few biopolymers but natSil-1A [23], which is isolated from diatoms, can serve above dual functions at the same time. Kroger [20] reported that natSil-1A could form the microscopic phase separation as template and induce silica-precipitating as catalyst, even in the absence of polyanions or multivalent anions. It can be explained that all the serine residues are phosphorylated and all the lysine residues are either methylated or covalently linked with polyamines. This structure ensures a highly zwitterionic polypeptide with the capability for self-assembly, which leads the microscopic phase separation driven by ionic interactions [23,24].

Inspired bynatSil-1A, we synthesized a novel dual functional polymer with zwitterionic structure. Specifically, alginate was chosen as the bulk polymer due to its long chain and abundant carboxyl group. Spermine was grafted onto alginate molecules through the oxidation-amination-reduction to obtain an aminated alginate (NH_2_-Alginate). Therefore, both the catalyzing function and templating function are incorporated into one polymer. Herein, sodium silicate is used as the precursor and mixed with NH_2_-Alginate. The feasibility of the nanocomposite for enzyme immobilization is demonstrated by using β-Glucuronidase (GUS) as the model enzyme.

## 2. Results and Discussion

### 2.1. The Catalyzing Function of NH_2_-Alginate

**Figure 1:**
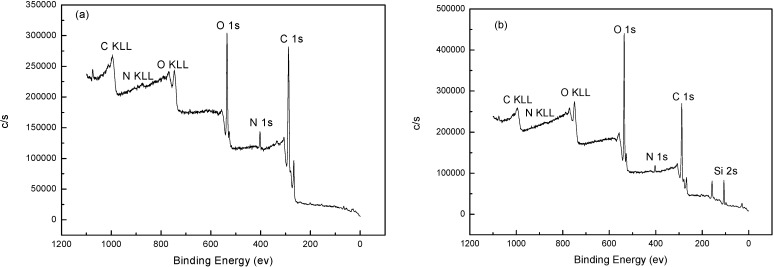
XPS spectra of NH_2_-Alginate (**a**) and NH_2_-Alginate/silica (**b**).

XPS spectra of NH_2_-Alginate and NH_2_-Alginate/silica nanocomposite are presented in Figure 1. A significant increase in nitrogen content demonstrates that the amine groups are successfully grafted onto the backbone of alginate. For the NH_2_-Alginate/silica, an intensive peak of element Si appears and demonstrates the successful biomimetic mineralization of silica. 

**Figure 2:**
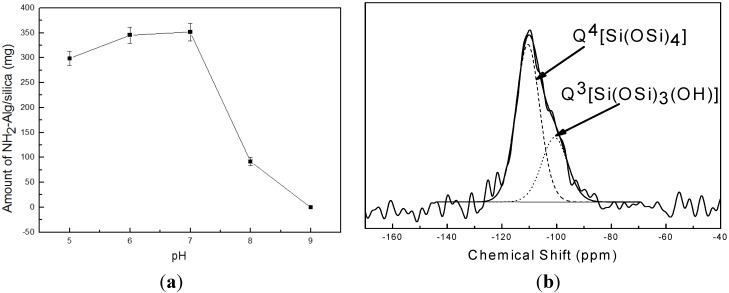
(**a**) The amount of NH_2_-Alginate/silica as a function of pH value; (**b**) NMR spectra of NH_2_-Alginate/silica.

To investigate the catalysis function of NH_2_-Alginate in silica precipitating, as shown in Figure 2a, the effect of pH value on the amount of NH_2_-Alginate/silica nanocomposite products is determined. It was reported that cationic proteins can attract anionic inorganic precursors through electrostatic and hydrogen bonding interactions, and then promoted hydrolysis and condensation of the precursors [16]. Similarly, NH_2_-Alginateis regarded as an acid-base catalyst with protonated amine group, which can induced the precursor hydrolysis and condensation. However, the amount of NH_2_-Alginate/silica nanocomposite sharply decreases if pH value is greater than 7.0. When pH value reached 9.0, little precipitate is observed due to decreasing positive charge on the surface of NH_2_-Alginate, which consequently weakens its electrostatic interaction with the precursor and then inhibits the catalysis function of NH_2_-Alginatein alkaline conditions. ^29^Si NMR is conducted to analyze the chemical structure and condensation degree of the mineralized silica catalyzed by NH_2_-Alginate. As shown in Figure 2b, the two main peaks near −100 ppm and −110.5 ppm are attributed to Q^3^ [Si(OSi)_3_(OH)] and Q^4^ [Si(OSi)_4_] with relative percentages of 30.2% and 69.8% respectively, which suggests the formation of well-condensed silica under the catalysis of NH_2_-Alginate. These results are in good agreement with the previous report that the mineralized silica using amine-containing polymers as catalysis and template exhibited a Q^4^/Q^3^ ratio from 1.0 to 2.5 [25,26,27,28,29].

### 2.2. The Template Function of NH_2_-Alginate

The average diameters of different NH_2_-Alginate micelles are characterized by DLS and the results are summarized in Table 1. Changing the ratio of [IO_4_^−^]/[alginate unit] to 1.0, the diameter decreases with the increasing molecular weight (*M*_w_) of amine agent, and the micelles diameter of 1#, 2#, 3# NH_2_-Alginate are 216.1 nm (1,2-ethylenediamine), 173.6 nm (Diethylenetriamine) and 145.0 nm (Spermine) respectively. A similar trend is also observed in lower ratio of 0.25 (4#, 5#, 6#) or 0.1 (7#, 8#, 9#). During the conjugation process, the number of amine group kept constant, consequentely, the molar concentration of amine agent with high *M*_w_ is lower. Therefore, the conjugating point on the backbone of alginate is less, which results in nonhomogeneous distribution of amine and forms small micelle template. When using the same amine agent, the micelles diameters decrease with the increasing of [IO_4_^−^]/[alginate unit] ratio (7# > 4# > 1#). Previous results showed that excess potassium periodate might induce further oxidation and break the main chain of alginate [30,31]. Therefore, NH_2_-Alginate with lower *M*_w_ leads to smaller micelles diameters.

**Figure 3:**
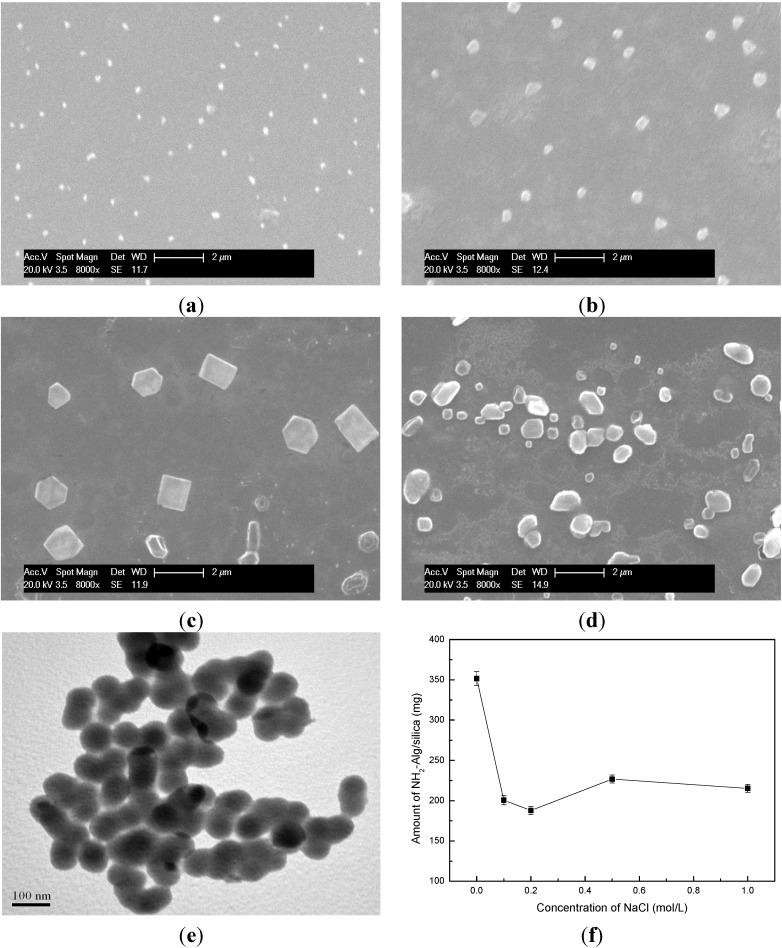
(**a**–**d**) SEM image of NH_2_-Alginate/silica; (**e**) TEM image of NH_2_-Alginate/silica; (**f**) the amount of NH_2_-Alginate/silica as a function of NaCl concentration.

The morphology of nanocomposite is presented in Figure 3. The size of resultant 3# (Figure 3a), 6# (Figure 3b), 8# (Figure 3c), 9# (Figure 3d) NH_2_-Alginate/silica nanocomposite is nearly 150 nm, 300 nm, 1000 nm, and 500 nm respectively, which is to the diameter of their related NH_2_-Alginate micelles. Previous researches showed that the silica-precipitating polymers could pre-self-assemble into different “shapes” or exhibit different sizes in solution, and then be used as templates to direct the final silica morphology [19,32]. This research shows that the micelles could be formed via microscopic phase separation and then displayed the template function for producing silica-based nanocomposite. The higher oxidation ratio and dense amine group may lead to small diameters of NH_2_-Alginate micelles and resultant nanocomposite (3#). In contrast, large Mw and long chain polymer could self-assemble and induce hexagonal [33] or square nanocomposite (8#). As 9# NH_2_-Alginate, nonhomogeneous distribution of amine may lead amorphous nanocomposite product, and the TEM images show that 9# NH_2_-Alginate/silica nanocomposite consists of nanoparticles with a diameter of 50 nm.

**Table 1:** The average diameter of different NH_2_-Alginate micelles.

No.	[IO_4_^−^]/[alginate unit]	Amination Agent	Diameter (nm)
1#	1	1,2-ethylenediamine	216.1
2#	1	Diethylenetriamine	173.6
3#	1	Spermine	145.0
4#	0.25	1,2-ethylenediamine	404.9
5#	0.25	Diethylenetriamine	344.5
6#	0.25	Spermine	331.9
7#	0.1	1,2-ethylenediamine	1535
8#	0.1	Diethylenetriamine	1160
9#	0.1	Spermine	598.7

It had been proved that multivalent anions were widely used for leading “microscopic phase separation”, while protein or polyamine was incapable of precipitating silica if replacing the multivalent ions with monovalent ions [21]. In this study, the effect of Cl^−^ on the formation of NH_2_-Alginate/silica nanocomposite is determined. About 350 mg of nanocomposite is obtained in deionized water, and nearly 200 mg is obtained upon preparing in NaCl solution. The zwitterionic structure of NH_2_-Alginate (polyamine moieties and carboxy groups) causes the self-assembly process via electrostatic interactions even in NaCl solution. However, the amine groups of NH_2_-Alginateare attracted by Cl^−^ ions, consequently, the electrostatic interaction is partly shielded and the microscopic phase separation is hindered. Therefore, the amount of NH_2_-Alginate/silica nanocomposite is decreased to some extent. 

### 2.3. Enzyme Activity Assay

Because excess potassium periodate might induce further oxidation and break the main chain of alginate in the synthesis of NH_2_-Alginate [30,31], it might induce loosing networks and a high release rate of enzyme if NH_2_-alginate individually formed hydrogel and encapsulated GUS. However, when it was used in biomimetic mineralization, the resultant silica presented dense network and confinement effect for the enzyme. In every enzymatic conversion process we measured the releasing GUS in the solution by the micro-Bradford method, but there was no GUS release detected. It is a significant advantage of biopolymer-inorganic nanocomposite.

**Figure 4:**
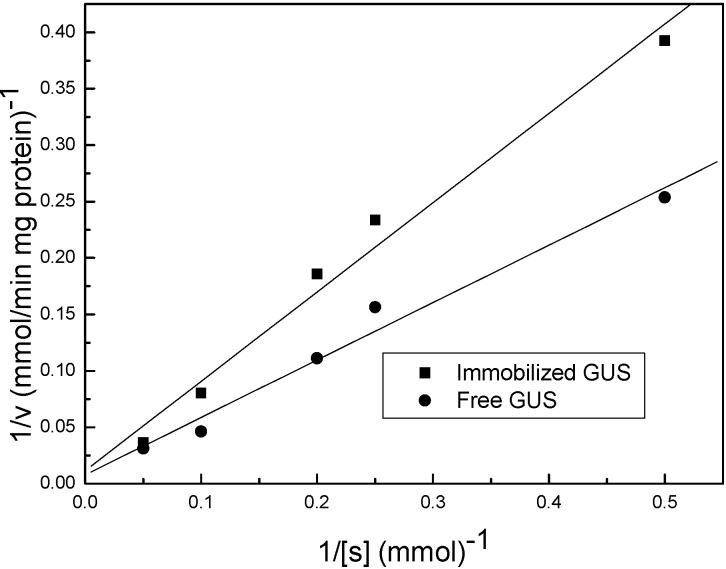
Typical lineweaver-Burk plots for free and immobilized GUS.

The enzymatic conversion of baicalin follows the Michaelis-Menten kinetics (Figure 4). The corresponding Michaelis constant (*K*_m_) and the maximum reaction rate (*V*_max_) are calculated according to the Lineweaver-Burk plots and presented in Table 2. Michaelis constant (*K*_m_) is an indicator for evaluating the binding ability between the substrate and enzymes, and usually increases after immobilization. However, in this study, the *K*_m_ for the immobilized GUS is increased slightly, indicating a well-preserved binding ability between GUS and the substrates [34]. The mesopores in the nanocomposite ensure the free motion of enzyme molecules, and the sufficient water in the nanocomposite enables enzyme molecules to catalyze in a nature-like microenvironment. While the maximum reaction rate (*V*_max_) decreases due to the increased diffusion resistance for substrate/product molecules [35].

**Figure 5:**
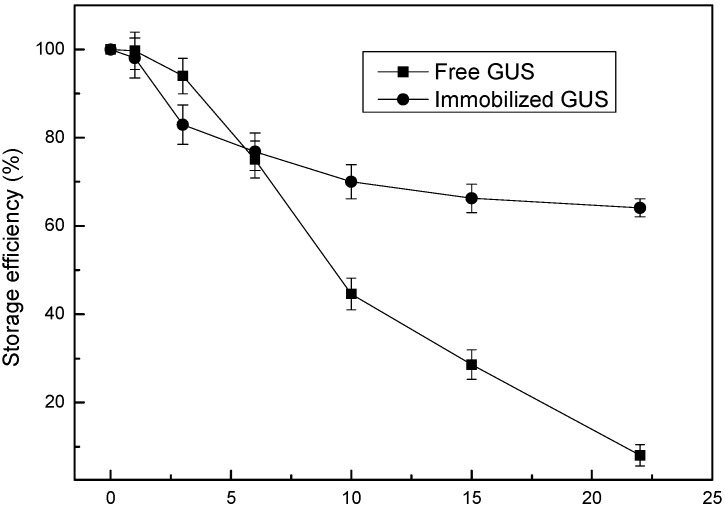
Storage stability of free and immobilized GUS.

The storage stabilities of free and immobilized GUS are compared in Figure 5. It should be noticed that the storage stability was obviously enhanced after immobilization. During the first six days, the activity of immobilized enzyme decreased synchronously with the free one (from 100% to ~75%). From 7th to 22nd day, the relative activity of free GUS decreased to 7%, while 65% activity of GUS immobilized in NH_2_-Alginate/silica nanocomposite was retained. This result is tentatively explained by the unfolding process, which is the main reason for enzyme deactivation during storage. It is supposed that in the initial storage time NH_2_-Alginate/silica nanocomposite may allow the unfolding to some extent like it happened in solution, since GUS is immobilized in a nature-like microenvironment. However, after a certain period of storage (six days in this case), the further unfolding of immobilized enzymes will be inhibited by the confinement of mesopores [35,36]. As a consequence, the decreased rate of activity for immobilized GUS become lower than its free form.

**Table 2:** Kinetic parameters for free and immobilized GUS.

GUS	*K*_m_ (mM)	*V*_max_ (μmol/min·mg GUS)
free	67.52	135.29
immobilized	69.33	91.57

**Figure 6:**
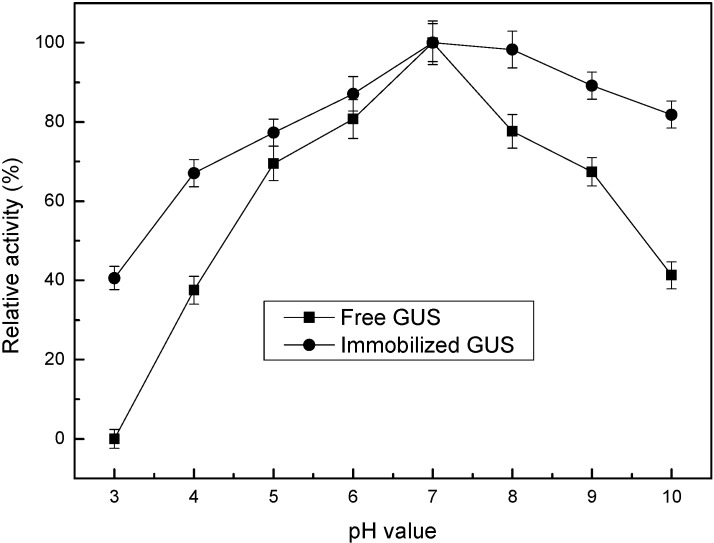
Effect of pH value on the activity of free and immobilized GUS.

Figure 6 shows the relative activity for free and immobilized GUS with the variation of pH values. The optimal pH value remained unchanged (pH 7.0) after immobilization in the NH_2_-Alginate/silica nanocomposite. The immobilized GUS keeps higher relative activity than its free form under extreme acidic conditions till pH 3.0 (85% *vs.* 60%) and alkaline conditions till pH 9.0 (40% *vs.* 0%), respectively. In general, the changes in pH would affect the charges carried by different amino acid residues of protein. Enzymes will undergo reversible or irreversible conformational changes and lose their activity under extreme pH conditions. Two kinds of functional groups, –NH_2_ and –COOH are distributed along the backbone. The functional groups can form abundant –COO^−^/–COOH, =NH/=NH_2_^+^ and –NH_2_/–NH_3_^+^ pairs, so it can tune the local pH value to some extent in case the bulk pH changed (the so-called buffering effect). In acidic medium, =NH, –NH_2_, and –COO^−^ can attract and consume the H^+^ ions, thus preventing H^+^ from diffusing into the inner polymer matrix and contacting the enzymes. On the contrary, in alkaline medium, =NH_2_^+^, –NH_3_^+^, and –COOH will release H^+^ ions, regulating the content of H^+^ inside the NH_2_-Alginate/silica nanocomposite. Thus, the zwitterionic structure of polymer provides above properties called “buffering effect”, which was also presented in our previous research about another type of NH_2_-alginate [37]. By this way, the pH change in the microenvironment is envisaged to be smaller than that happening in the bulk solution [38,39]. Additionally, several studies also had reported that the immobilized enzymes exhibited higher relative activity than their free form in extreme acidic conditions and alkaline conditions, and mainly attributed that to “confinement effect” of enzymes in mesoporous silica, which leads to the restricted conformational changes under extreme pH conditions [40,41,42]. Therefore, the above two effects act synergistically so that the extreme pH tolerance was enhanced.

## 3. Experimental Section

### 3.1. Materials

GUS (EC 3.2.1.31) from *Escherichia coli* (type IX-A, lyophilized powder, 1,000,000–5,000,000 units/g protein) and Sodium alginate were obtained from Sigma Chemical Co. (St. Louis, MO, USA). Spermine, sodium periodate and sodium borohydride were obtained from Fluka Chemie (Buchs, Switzerland). All of other solvents and reagents were of analytical grade.

### 3.2. Synthesis of NH_2_-Alginate

**Figure 7:**
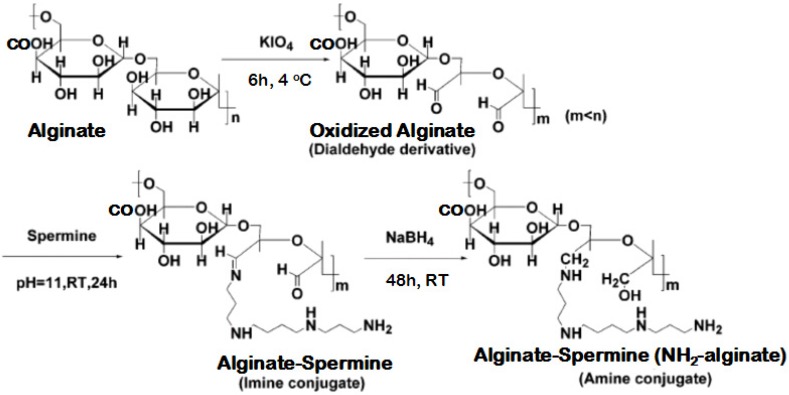
Illustration of NH_2_-Alginate synthesis process.

Preparing NH_2_-alginate is similar with the process of dextran bioconjugation, which was described in previous research by Domb’s group [43]. The scheme is presented in Figure 7.

#### 3.2.1. Oxidation

Alginate (10 g, 50.5 mmol of units) was dissolved in 200 mL of double deionized water (DDW). Potassium periodate at a mole ratio (IO_4_^−^/alginate units) from 0.1 to 1.0 was separately added to this solution and the mixture was vigorously stirred in the dark at 4 °C until a clear yellow solution was obtained (12 h). The unreacted periodate (IO_4_^−^) was removed by adding ethylene glycol, followed by extensive dialysis against DDW (12,000 cut-off cellulose tubing) for 2 days and at 4 °C. Purified oxidized alginate were freeze-dried to obtain a white powder. 

#### 3.2.2. Amination

Amination agent containing 63.125 mmol amine group was dissolved in 50 mL of borate buffer (0.1 M, pH 11). A solution of oxidized alginate in 100 mL of DDW was slowly added during 5 h (sage metering pump) into the aminnation agent solution. The mixture was gently stirred at room temperature for 24 h and dialyzed against DDW at 4 °C, applying 3500 cutoff cellulose tubing (Membrane Filtration Products, Inc., San Antonio, TX, USA). The imine-based conjugate was obtained by following lyophilization. 

#### 3.2.3. Reduction

The amine based conjugates (reduced) were obtained after reducing the imine conjugates with excess 1 g NaBH_4_ in water at room temperature for 24 h.The reduction was repeated with additional portion of NaBH_4_ (1 g) and stirring for 24 h at the same conditions. Then the resulting light-yellow solution was dialyzed against DDW at 4 °C for 2 days followed by freeze-drying to obtain amined alginate (NH_2_-Alginate).

### 3.3. Preparation of NH_2_-Alginate/Silica Nanocomposite and Encapsulation of GUS

Nanocomposite preparation: NH_2_-Alginate was suspended in DDW or NaCl solution (10 mg/mL). A freshly prepared 100 mmol/L sodium silicate solution was obtained by dissolving sodium silicate in water followed by acidification to a specific pH with HCl. The NH_2_-Alginate solution (20 mL) was then mixed with 30 mL sodium silicate solution, and allowed to react for 10 min. The resultant precipitates were collected by centrifugation, rinsed twice with deionized water to remove unreacted agent, and lyophilized to dryness. 

GUS encapsulation: The GUS could be dissolved in NH_2_-Alginate solution and immobilized in a co-pericipitating process. A mixture of NH_2_-Alginate (10 mg/mL) and GUS (0.05 mg/mL) was prepared by dissolving them in Tris-HCl buffer solution (pH 7.0, 30 mmol/L). Freshly prepared 100 mmol/L sodium silicate solution was obtained by dissolving sodium silicate in water followed by anacidification to pH 7.0 with HCl. 20 mL of NH_2_-Alginate/GUS solution was then added to 30 mL of sodium silicate solution. After 10 minutes, the resultant precipitates were centrifuged and rinsed twice with deionized water to remove residual GUS, NH_2_-Alginate and silicate. The supernatant was collected and the GUS content was determined by the Bradford method using a UV spectrophotometer (U-2800, Hitachi, Tokyo, Japan). The encapsulation efficiency was calculated according to Equation (1):

(1) $$\text{Encapsulation efficiency (\%) = }\left(1-\frac{C_{1}V_{1}}{C_{0}V_{0}}\right)\times 100$$

where *C*_0_ (mg·mL^−1^) and *V*_0_ (mL) were the concentration and volume of introduced GUS solution, respectively; *C*_0_*V*_0_ (mg) was the introduced GUS amount in the immobilization medium; *C*_1_ (mg·mL^−1^) and *V*_1_ (mL) were the GUS concentration and supernatant volume when preparing GUS-containing nanocomposite, respectively; *C*_1_*V*_1_ (mg) was the amount of GUS leaked out during the preparation process of GUS-containing nanocomposite.

### 3.4. Characterizations

The surface properties of microcapsules were characterized by X-ray photoelectron spectroscopy (XPS) in a Perkin-Elmer PHI 1600 ESCA system with a monochromatic Mg *K*α source and a charge neutralizer. 

Solid-state ^29^Si MAS NMR spectra of the NH_2_-Alginate/silica nanocomposite were recorded on an Infinity Plus-300 MHz spectrometer (Varian, CA, USA) with resonance frequencies of 59.63 MHz for ^29^Si. The magnetic field was 7.05 T, and the spin rate of the sample was spun at 3 kHz. 

For Scanning electron microscopy (SEM) analysis, samples were prepared by applying a drop of the particle suspension to a glass slide and then drying overnight. After that, the samples were sputtered with gold. Measurements were conducted using Philips XL30 ESEM and Hitachi S-4800 instrument (Hitachi, Tokyo, Japan) at an operation voltage of 20.0 keV and 0.7 keV. 

Transmission electron microscope (TEM) observation was performed on a JEM-100CXII instrument (JEOL, Tokyo, Japan).

The average size of NH_2_-Alginate micelles was measured by BrookhavenInstruments BI200SM dynamic light scattering (DLS) system. All samples was dispersed in 0.05 M Tris-HCl buffer solution (pH 7.0).

### 3.5. Enzyme Activity Assay

Bioconversion of baicalin to baicalein is catalyzed by GUS, which had been described in our previous research [44]. Free or immobilized GUS was introduced into a beaker containing 20 mL of 0.09 mM baicalin and 0.1% w/v Na_2_SO_3_, both dissolved in 30 mM Tris-HCl buffer. The beaker was sealed and the reaction was performed under stirring. After 60 min, 100 μL of the reacting solution was sampled and analyzed by HPLC (HP1100, Agilent, CA, USA). The enzyme activities and stabilities were studied and compared by measuring the amount of baicalein produced, and each data point was replicated three times.

The optimum conditions for GUS activity were determined by testing the enzyme activity under a range of pH values (3–10). The activity of GUS was calculated based on the amount of baicalein produced and was expressed as relative activity compared with the activity at the optimum pH.

GUS immobilized in NH_2_-Alginate/silica nanocomposite was stored at 4 °C for a certain period of time. The storage stability was compared in terms of storage efficiency which was defined as the ratio of immobilized enzyme activity after storage to their initial activity.
